# Supplementary material for: Esrrb extinction triggers dismantling of naïve pluripotency and marks commitment to differentiation
Source: EMBO J. 2018 Oct 1;37(21):e95476. doi: 10.15252/embj.201695476 (PMC6213284; doi:10.15252/embj.201695476)
Supplement: Supplementary file 1 — Appendix [file EMBJ-37-e95476-s001.docx]

**Festuccia et al. - Appendix**

**Table of Contents**

**Appendix Figures: … Pages 2-4**

**Appendix and Expanded View Figure Legends: … Pages 5-7**

**Appendix Table 1 - External datasets: … Page 8**

**Appendix Supplementary Methods: … Pages 9-16**

**Appendix Supplementary References: … Pages 17-19**

**
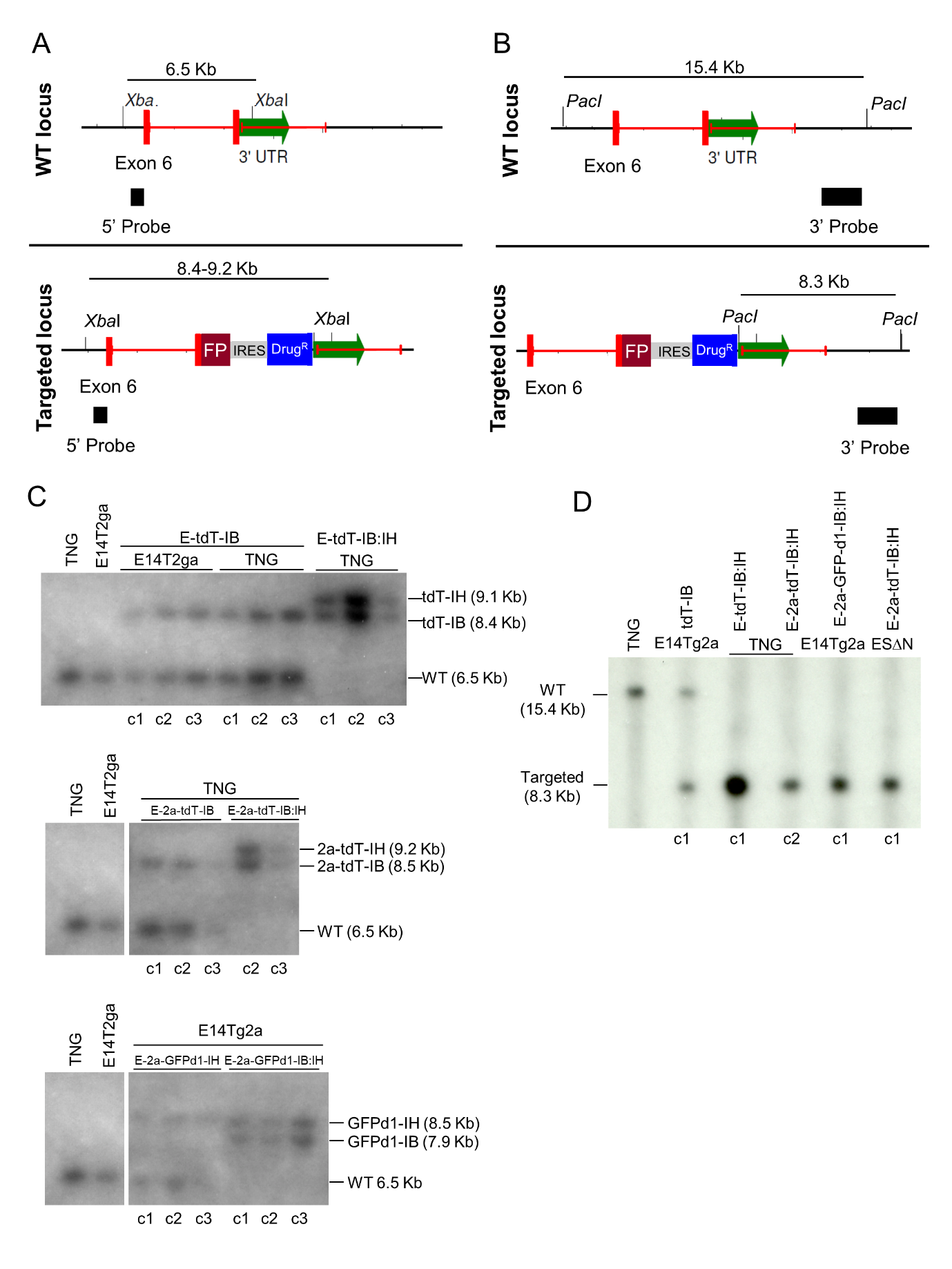
**

**Appendix Figure S1**

**
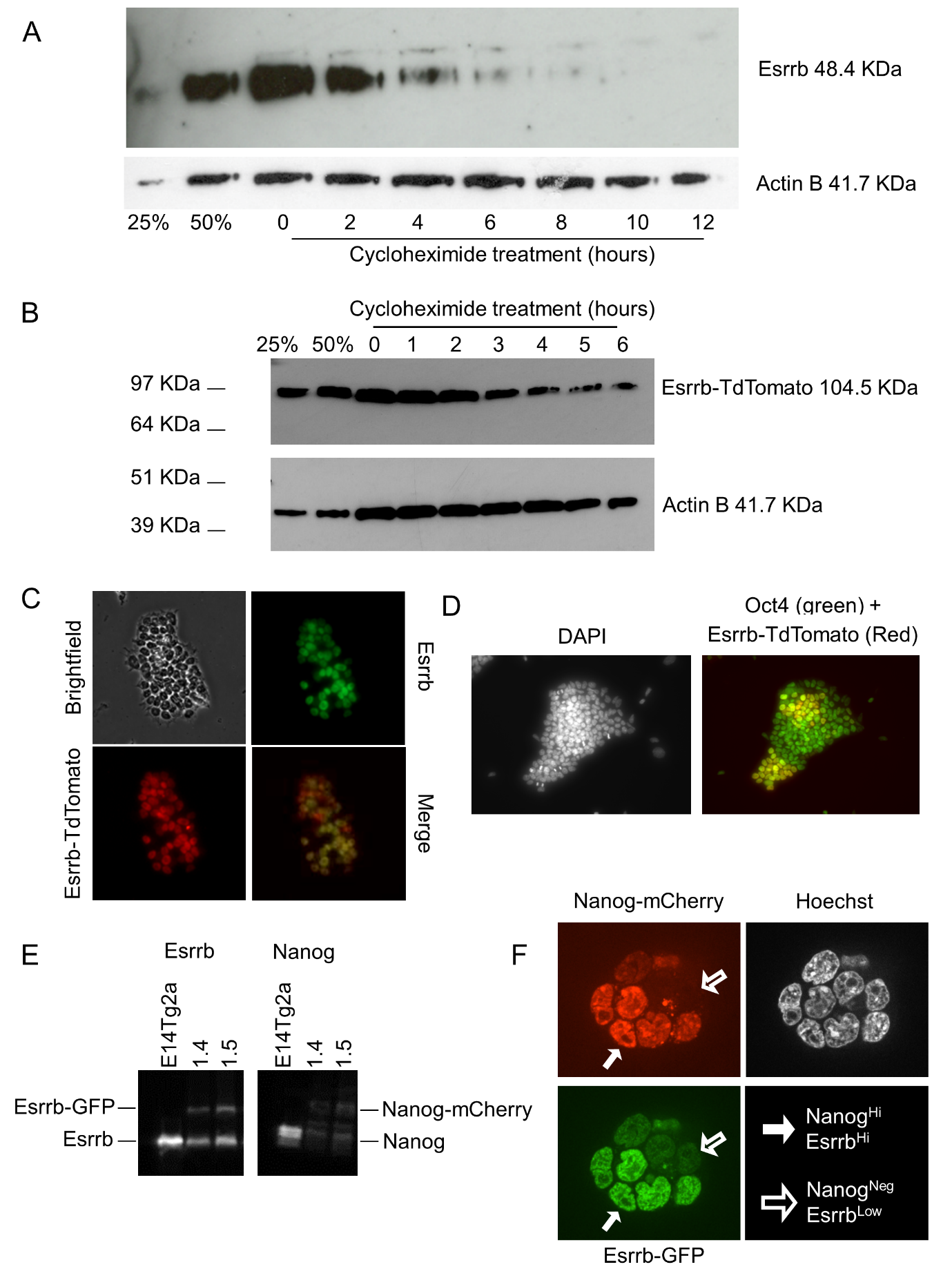
**

**Appendix Figure S2**

**
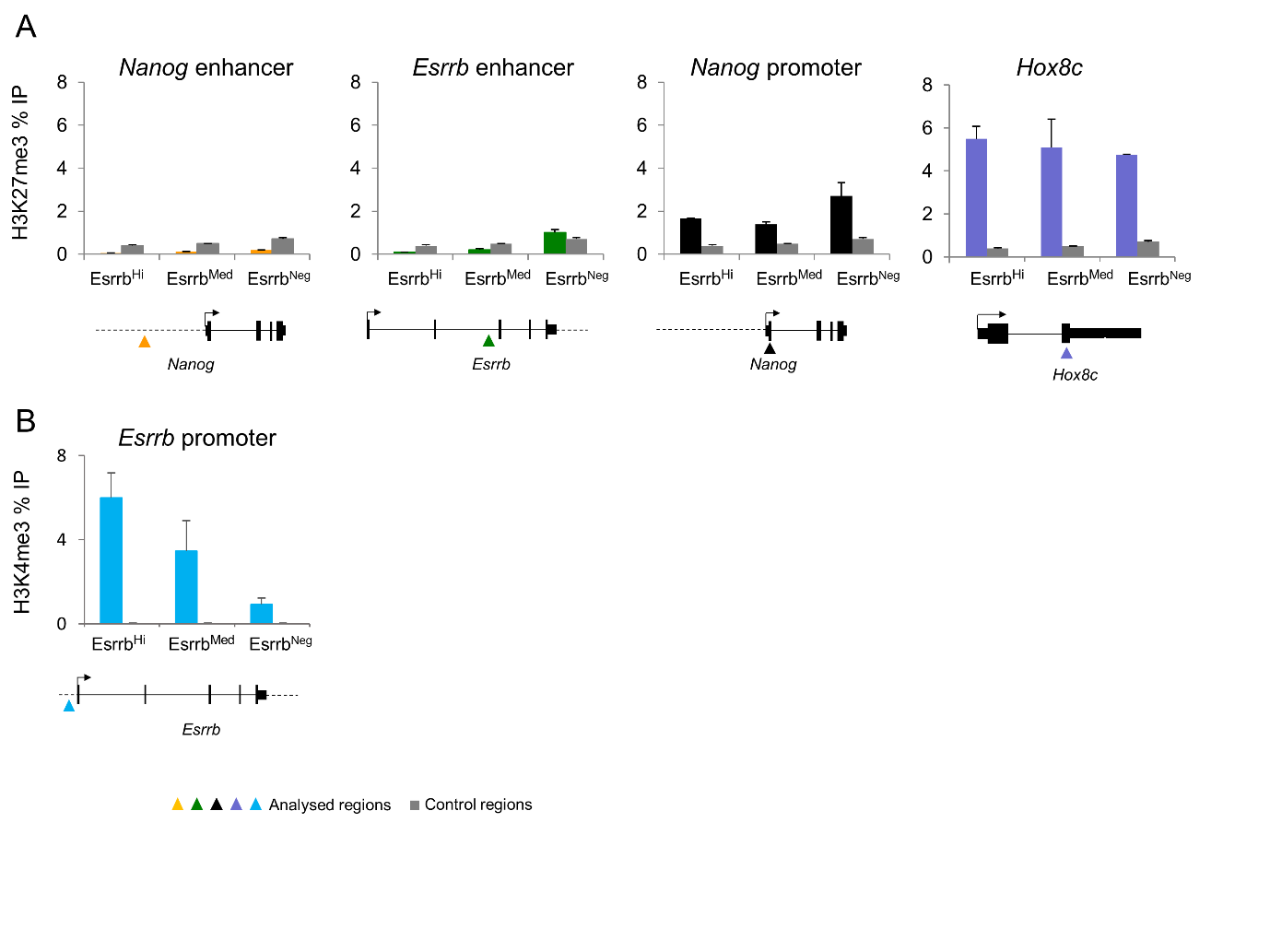
**

**Appendix Figure S3**

**Appendix Figure Legends:**

**Appendix Figure S1. Southern blot analysis of all ES cell lines derived in this study.**

**A, B**: Schematic representation of the 3’ end of the *Esrrb* gene showing exons 6 and 7 (Genome Reference Consortium Mouse Build 38/ mm10: chr12:86,512,000-86,533,000) before and after targeting. 5’ and 3’ homology arms are red lines, coding exons are red boxes, 3’UTR is green, fluorescent proteins (FP) are maroon, IRES is grey and DrugR (either *hph* or BSD^R^) is blue. FPs are linked to the *Esrrb* ORF by direct protein fusion or via a 2a sequence. Restriction sites and probes used for Southern blot analysis using 5’ (A) and 3’ (B) probes are indicated, along with the expected sizes of the DNA fragments obtained after *Xba* I or *Pac* I digestion of wild-type and TdTomato-IRES-Drug^R^ targeted alleles.

**C, D**: Southern blot of DNA from the indicated cell lines. DNA was digested with *XbaI* and analysed with the 5’ probe (C) or *PacI* and analysed with the 3’ probe (D). Expected fragment sizes relative to the targeting of the Esrrb-TdTomato fusion protein (E-tdT) or 2a linkages of TdTomato (E-2a-tdT) and GFP-d1 (E-2a-GFPd1) cassettes in combination with IRES-Hygromycin (iH) or IRES-Blasticidin (iB) resistance genes are indicated.

Relates to all figures.

**Appendix Figure S2. Characterisation of Esrrb-TdTomato and NER reporter lines.**

**A,** **B:** ESRRB and Esrrb-TdTomato protein half-lives in ESCs. E14Tg2a (A) or E-tdT (B) ESCs were treated with cycloheximide for the indicated times and analysed by immunoblotting for ESRRB and ACTIN B.

**C, D**: Immunofluorescence analysis of ESCs cultured for 3 days in GMEMβ/FCS/LIF without selection. **C,** E-tdT ES cells were analysed for ESRRB (top, right) or fluorescence from Esrrb-TdTomato (bottom, left). **D**: OCT4 expression and fluorescence from Esrrb-tdTomato in TNG E–tdT ESCs. Note that Esrrb^negative^ cells retain OCT4 expression.

**E:** Western blot showing the expression of both endogenous and -GFP or -mCherry tagged ESRRB and NANOG proteins in NER ESCs. Clone 1.5 was used in subsequent experiments.

**F:** Live spinning-disk confocal imaging of NER ESCs released from Puromycin and Balsticidin selection in GMEMβ/FCS/LIF for three days. Hoechst was added to the culture medium 20 minutes before imaging. The solid arrows indicate cells expressing high levels of both Nanog-mCherry and Esrrb-GFP. Empty arrows denote cells negative for Nanog-mCherry but still expressing detectable levels of Esrrb-GFP.

Relates to Figures 1 and 2. Data information: For a schematic representation of the reporter allele configuration characteristic of each cell line please refer to Fig EV1.

**Appendix Figure S3. H3K27me3 does not substantially accumulate at promoters and enhancers of pluripotency genes in Esrrb^Neg^ cells, but H3K4me3 is lost from the *Esrrb* promoter.**

**A)** Quantitative ChIP-PCR analysis of H3K27me3 enrichment at the *Esrrb* and *Nanog* enhancers or the *Nanog* promoter in sorted SSEA-1^+^ / Esrrb^Hi^, Esrrb^Med^ or Esrrb^Neg^ E-GFPd1 ESCs. High H3K27me3 enrichment at the *Hox8c* locus is shown as a positive control. Error bars: standard deviation of the measures in two independent ChIP experiments, each performed on pooled chromatin from at least three independently sorted samples. The diagrams at the bottom show the relative position of the regions analysed for enrichment of histone modifications (in colour or black). Enrichment at control genomic locations is shown in grey.

**B)** Quantitative ChIP-PCR analysis of H3K4me3 enrichment at the *Esrrb* promoter in sorted SSEA-1^+^ / Esrrb^Hi^, Esrrb^Med^ or Esrrb^Neg^ E-GFPd1 ESCs. Error bars: standard deviation of the measures in three independent ChIP experiments, each performed on pooled chromatin from at least three independently sorted samples. The diagram shows the relative position of the regions analysed for enrichment of histone modifications (in colour or black). Enrichment at control genomic locations is shown in grey.

Relates to Figure 4.

| Description | Original Publication(s) |
| --- | --- |
| ChIP-seq data for IgG in E14 mouse ESCs (control data) | ([Ho et al., 2009](#_ENREF_8)), ([Wu et al., 2011](#_ENREF_18)), ([Percharde et al., 2012](#_ENREF_14)) |
| Microarray, DeepSAGE and ChIP-seq from our previous publication | ([Festuccia et al., 2012](#_ENREF_6)) |
| ChIP-seq data for TCFCP2L1, EP300, E2F1, NANOG, SUZ12, ESRRB, CTCF, SOX2, OCT4, KLF4 | ([Chen et al., 2008](#_ENREF_4)) |
| ChIP-seq data for EP300, OCT4, OTX2, H3K27ac, H3K4me1, and FAIRE-seq data | ([Buecker et al., 2014](#_ENREF_1)) |
| ChIP-seq data for MED1 | ([Kagey et al., 2010](#_ENREF_12)) |
| ChIP-seq data for CHD7 | ([Schnetz et al., 2010](#_ENREF_17)) |
| ChIP-seq data for PRDM14 | ([Ma et al., 2011](#_ENREF_13)) |
| ChIP-seq data for OTX2, H3K27ac, H3K4me1, OCT4, and FAIRE-seq data | ([Yang et al., 2014](#_ENREF_19)) |
| ChIP-seq data for FOXD3 | ([Respuela et al., 2016](#_ENREF_15)) |

**Appendix Table 1**. Overview of data used for meta-analyses.

**Supplemental Methods**

**ESC genomic DNA isolation and Southern Blot analysis**

ESCs were grown in gelatinised 25cm^2^ flasks (IWAKI Cat # 3100-025) until reaching 70-80% confluence. Cells were collected by trypsinisation and washed once in PBS. Genomic DNA was prepared from cell pellets using the DNeasy kit (Qiagen Cat # 69504) following the manufacturer’s instructions and performing the elution step twice with 100μl of nuclease free water pre-warmed at 42^o^C. 4μg of genomic DNA were digested overnight with the indicated restriction endonucleases. Digested DNA was separated by electrophoresis on a 0.8% w/v agarose gel and the gel incubated for 30 min in a water solution of 1μg/ml ethidium bromide. After crosslinking for 2 min by exposure to 254 nm wavelength UV, the gel was incubated twice for 15 min in a 0.5M NaOH, 1M NaCl solution and washed in a 0.5M Tris, 3M NaCl pH 7.4 solution. DNA was wet transferred to a Hybond XL membrane (General Healthcare Cat # RPN303-S) soaked in 2X SSC (20X SSC: 3M NaCl, 0.3M Tri-sodium Citrate) by capillary flow of a 20X SSC solution. The membrane was washed in 2X SSC, dried and baked for 2 hours at 80°C. The membrane was blocked with PerfectHyb solution (Sigma Cat # H7033) containing 100μg/ml salmon sperm DNA (Sigma Cat # D7656) at 68°C in a roller bottle. 25ng of probe DNA were labeled with P^32^ α-dCTP using a Rediprime II Random Prime Labeling Kit (Amersham Cat # RPN1633). The membrane was hybridised with the labelled probe overnight at 68°C, rinsed, and washed twice in 0.5 SSC 0.1%SDS for 90 and 30 min respectively at 68°C. The membrane was exposed to Hyperfilm (Amersham Cat # 28906837) at -80C for 1-7 days depending on the signal intensity.

**Immunoblotting**

E14Tg2a Esrrb-2a-GFPd-IBIH ESCs were plated (3000/cm^2^) and cultured in the absence of selection for 3 days. Cells were then harvested by trypsinisation, and resuspended at 2 x 10^6^ cells/ml in 10%FCS/PBS. Keeping the samples refrigerated, Esrrb-GFP^high^ (Top 10%), Esrrb-GFP^medium^ (15% of the distribution immediately above negative) and Esrrb-GFP^negative^ cells were purified using a FacsARIA cell sorter (Becton, Dickinson). Cells were lysed in 300 μl 0.5% NP-40, 50mM Tris pH 8,150mM NaCl containing protease inhibitors (Roche Cat # 04 693 116 001). Protein extracts were treated with 2 μl Benzonase (Novagen, Cat # 70664-3) (1 h, 4°C). 50 μg of protein was denatured in Laemmli buffer (100°C, 5 min) and fractionated on a NuPage-Novex 10% Bis-Tris gels (Invitrogen, Cat # NP0302). Proteins were electro-blotted to a nitrocellulose membrane in 25mM Tris, 0.21 M glycine, 20% methanol. The membrane was blocked in PBS/0.01% Tween (PBST), 10% non-fat dry milk for 2 h and incubated in 5ml of PBST 5% non-fat dry milk containing mouse monoclonal anti-HDAC-2 (Upstate, Cat #05-814), mouse monoclonal anti-ESRRB (Persaeus Proteomics, Cat # PP-H6707-00) (5 μg) and anti-NANOG rabbit polyclonal (Chambers, 2007) (4.4 μg) antibodies or goat polyclonal anti-OCT4 (Santa Cruz Biotechnology, Cat # SC-8628) (1 μg) (overnight, 4°C). Membranes were washed 3 times for 20 min in PBST and incubated with li-cor anti-mouse IgG (Cat # 92668072) and anti-rabbit IgG (Cat # 92632213) conjugated or anti-goat IgG (Cat # 92668074) fluorescent dye conjugated secondary antibodies. Membranes were washed in PBST and signal detected on a LI-COR Odyssey.

**Immunofluorescence on cultured cells for timecourse experiments.**

3x10^5^, 10^5^ and 2x10^4^ E14Tg2a cells were plated in one section each of Ibidi 4 section dishes (Ibidi, µ-Dish 35 mm, high, cat. 81156) 1, 2 and 3 days before fixation respectively and cultured in GMEMβ/FCS/LIF. 2x10^4^ E14Tg2a cells were plated in the fourth well in N2B27/2i/LIF 3 days before fixation. In parallel, as a negative control, 1x10^5^ cells were plated in a separate IBIDi plate in GMEMβ/FCS without LIF and in the presence of 10^-6^ M retinoic acid. Cells were fixed on day 3 in PBS/4% PFA for 10 min at RT. Cells were permeabilised with PBS/0.1% v/v TritonX100 (15 min, RT). Blocking was performed (30 min, RT) in PBS/0.1% v/v TritonX100 supplemented with 3% of donkey serum (blocking buffer). Primary antibodies (diluted in blocking buffer to the concentrations indicated below) were applied (1-2 hours, RT or overnight at 4°C) in a volume of 2ml per dish. Dishes were modified to ensure flow of the solution between sections, and avoid differences in staining levels among different conditions. After three washes (5min RT) in PBS/0.1% v/v TritonX100, secondary antibodies (indicated below, diluted to 2μg/ml in blocking buffer) were applied (1 hour, RT). Cells were washed at least three times (5min RT) in PBS/0.1% v/v TritonX100 and nuclei counterstained with 4',6-diamidino-2-phenylindole (DAPI). Primary antibodies were used at the following concentrations: anti-NANOG rabbit polyclonal (Cosmobio, Cat. REC-RCAB001P) 0.4ug/ml (1:500); anti-OCT4 (Santa Cruz Biotechnology, Cat # sc-5279), 0.4μg/ml; anti-ESRRB (Persaeus Proteomics, PP-H6705-00), 2 μg/ml. Secondary antibodies: Alexa Fluor 488 AffiniPure Donkey Anti-Rabbit IgG (H+L) (Jackson ImmunoResearch, Cat. 711-545-152); Alexa Fluor 594 AffiniPure Donkey Anti-Mouse IgG (H+L) (Jackson ImmunoResearch, Cat. 715-585-150); Alexa Fluor 647 AffiniPure Donkey Anti-Goat IgG (H+L) (Jackson ImmunoResearch, Cat. 705-605-003); Imaging was performed on a LSM800 Zeiss microscope, and images analysed with CellProfiler 2.1.1 ([Carpenter et al., 2006](#_ENREF_2)). Briefly, cell nuclei were identified on the DAPI images, using an automatic thresholding strategy based on object shape, and the mean intensity value of each nucleus calculated for the OCT4, NANOG and ESRRB immunofluorescence images. Data was exported and plotted using the Dataframes, Plots and PlotlyJS packages in Julia (https://julialang.org/). Histograms were plotted in Julia using the Plots, PlotlyJS, StatPlots, KernelDenisty, and DataFrames packages.

**Chromatin immunoprecipitation (ChIP)**

For each experiment a total of 5-10 μg of chromatin was used, pooling material from at least three independent preparations. Chromatin was thawed and pre-cleared (90 min rotating on-wheel, 4°C) in 1 ml TSE/150mM NaCl containing 50µl of a 50% slurry of pA/pG sepharose beads (Sigma Cat # P9424-5ML, P3296-5ML), previously blocked with 500µg/ml BSA (Roche, Cat # 5931665103) and 1µg/ml of yeast tRNA (Invitrogen, Cat # AM7119). Pre-cleared chromatin was centrifuged (3000rpm, 1 min) in a benchtop microcentrifuge and the supernatant transferred into fresh tubes. 10μl of diluted chromatin was set apart for input DNA extraction and precipitation. Immunoprecipitations with anti-NANOG rabbit polyclonal (0.5 μg/IP) ([Chambers, 2004](#_ENREF_3)), anti-ESRRB mouse monoclonal (Euromedex, Cat # PB-7C2) (1 μg/IP), goat polyclonal anti-OCT4 (Santa Cruz Biotechnology, Cat # SC-8628) (1 μg/IP), rabbit polyclonal anti-H3K4me1 (Abcam, Cat # Ab8895), rabbit polyclonal anti-H3K27ac (Active Motif, Cat # 39133), rabbit monoclonal anti-H3K4me1 (Cell Signalling, Cat #9751S), and rabbit monoclonal anti-H3K27me3 (Cell Signalling, Cat #9733S) antibodies were performed by rotating on-wheel (4°C, overnight) in a final volume of 500µl TSE/150mM NaCl. Immunocomplexes were recovered by rotating (4h, 4°C) with 25ul of a 50% slurry of pA/pG sepharose beads (Sigma Cat # P9424-5ML, P3296-5ML) or 20µl pA/pG magnetic Dynabeads (Life Technologies Cat # 10001D, 10003D) (30 mg/ml), (blocked as above). Beads were recovered by centrifugation or magnetic separation and washed sequentially (5 min rotation, RT) with 1ml of the following buffers: TSE/150mM NaCl (3x), TSE/500mM NaCl (once), TSE/750mM NaCl (once), TSE/1000mM NaCl (once), washing buffer (10mM Tris-HCl pH8, 0.25M LiCl, 0.5% NP40, 0.5% Na-Deoxycholate, 1mM EDTA), and twice in TE (10mM Tris-HCl pH8, 1mM EDTA). After the last wash, a 2-step elution was performed by incubating beads in 150µl 1% SDS, 10mM EDTA, 50mM Tris-HCl, pH8 (15 min; 65°C) after vigorous vortexing. Eluates were collected (1 min, 14,000 rpm), a second elution performed under the same conditions and both eluates pooled. For both immunoprecipitated and input chromatin samples, crosslinking was reversed by incubation (65°C, overnight) followed by proteinase K treatment (5 μl - Invitrogen Cat # AM2546), phenol/chlorophorm extraction and ethanol precipitation using 40μg of glycogen as a carrier. DNA was resuspended in 60µl H_2_O.

**Library preparation and sequencing**

Material from three independent ChIPs was pooled, resuspended in 10μl H_2_O and used as a template for library preparation using a MicroPlex Library Preparation Kit (Diagenode Cat # C05010010) following the manufacturer instructions. The library preparation and synthesis steps were performed using a benchtop thermocycler (Biorad). For both OCT4 and NANOG ChIP libraries Esrrb-GFP^high^, Esrrb-GFP^medium^ and Esrrb-GFP^negative^ samples were labelled with indexing reagents n. 4, 6 and 12 respectively. Before library amplification, Picogreen (Life Technologies Cat # P11496) was added at working concentration (1:200 from stock) in the amplification mix. 75μl of mix for each sample was transferred to three wells of a 384 well qPCR plate (Roche) and library amplification performed using a 480 LightCycler (Roche). Amplifications were stopped before the reaction reached plateau and DNA concentration quantified from 1μl of amplification reaction diluted 1:200 in TE based on a λ DNA standard curve using Picogreen (Life Technologies Cat # P7581) and a microplate fluorescence reader. In case the total amount of DNA was below 500ng, 4 additional cycles of amplification were performed. Amplified material was purified using Agencourt AMPure® XP magnetic beads (Beckman Coulter, Cat # No. A63880) and eluted in 30μl TE. Samples were submitted to the GenePool core facilities (University of Edinburgh) for quantification and analysis of fragment size on an Agilent 2100 Bioanalyser. Fragment sizes ranged from 200-450 bp in all samples. Equimolar amounts of OCT4 or NANOG ChIP libraries from Esrrb-GFP^high^, Esrrb-GFP^medium^ and Esrrb-GFP^negative^ sorted cells were pooled and sequenced on a single Illumina HiSeq 2500 flowcell.

**Quantification of transcription factor expression in single cells by flow cytometry.**

10^6^ cells were resuspended and fixed in 2 ml 0.25% PFA/PBS (1h, RT) on a tube roller. After 1 wash (5ml PBS), cells were resuspended in 2 ml 70% v/v methanol/PBS pre-cooled to 4°C and incubated (1 hour, 4°C) on a rotating wheel. After centrifugation, cells were resuspended in 1ml PBS/5% donkey serum and 100µl (10^5^ cells) transferred into single wells of a 96 well/V-bottom microtitre plate. Cells were centrifuged (450g, 3 min) and resuspended in 100μl of staining buffer (PBS, 1% BSA, 3mg/ml PVP, 0.1% TritonX-100, 10% donkey serum) pre-cooled to 4°C. After incubating (30 min, RT) cells were centrifuged and resuspended in 100µl of pre-cooled staining buffer containing goat polyclonal anti-OCT4 (Santa Cruz Biotechnology, Cat # SC-8628) and mouse monoclonal anti-KLF4 (Abcam, Cat # Ab75486) antibodies at final concentrations of 1.25µg/ml and 10µg/ml, respectively. Plates were incubated overnight (4°C) and washed (3x, 20 min, 4°C) in 200µl of pre-cooled staining buffer. After centrifugation, cells were resuspended in 100µl of pre-cooled staining buffer containing donkey anti-mouse IgG (H+L) Alexa fluor-647 (Invitrogen, Molecular Probes Cat # A-31571) and donkey anti-goat IgG Biotin conjugated (Millipore, AP180B) antibodies at a final concentration of 4µg/ml. Cells were incubated (2 hours, RT) in the dark and washed (3x, 20 min, 4°C) in 200µl of pre-cooled staining buffer. After centrifugation, cells were resuspended in 100µl pre-cooled staining buffer containing Streptavidin Alexa fluor-405 1µg/ml (Invitrogen, Molecular Probes Cat # S32351), centrifuged, washed (1x, 20 min, 4°C) in 200µl of pre-cooled staining buffer, centrifuged, resuspended in 200µl of PBS/5% donkey serum and analysed using a LSR II Fortessa flow-cytometer system (Becton, Dickinson). Data was analysed using the FlowJo software suite (Tree Star). To set the appropriate gates for KLF4 and OCT4 negative cells, RA-differentiated E14Tg2a cells were stained in parallel. For the analysis of KLF4, Esrrb-TdTomato and Nanog:GFP heterogeneity only OCT4^positive^ cells representing the undifferentiated population were taken into account.

**RNA isolation and quantitative real-time RT-PCR**

Total RNA from cultured cells or cells purified using a FacsARIA cell sorter (Becton, Dickinson) was isolated using the RNeasy minikit (Qiagen Cat # 74104) with on-column DNase I digestion (Qiagen Cat # 79254). Reverse transcription was performed on 1-2μg total RNA in a final volume of 20µl with 100U of SuperScriptIII (Invitrogen Cat # 18080-093), 200ng random hexamers (Invitrogen Cat # N8080127) at 42°C (60 min). Real-time RT**–**PCR was performed in triplicate in 384-wells plates with a 480 LightCycler (Roche) using LightCycler 480 SYBR Green I Master (Roche Cat # 04707516001) and 5μl cDNA per reaction. Standard curves of all primers were performed to check for efficient amplification (above 85%) and all melting curves were generated to verify production of single DNA species with each primer pair. PCR primer sequences are listed in Table EV4. Values for gene expression were normalised to the levels of TATA box Binding Protein (Tbp) or Tubulin b (Tubb).

**Microarray analysis**

E14Tg2a Esrrb-2a-GFPd-IBIH ESCs were plated at low density (3000/cm^2^), cultured for 3 days and sorted as described. RNA was prepared from at least 10^6^ sorted cells for each population and TNG Esrrb-2a-TdTomato EpiSC using a RNeasy minikit (Qiagen Cat # 74104). 100ng of RNA was reverse transcribed into double stranded cDNA and transcribed/amplified into biotin labelled cRNA using an Illumina TotalPrep RNA Amplification Kit (Ambion Cat # AMIL1791). Labelled RNA was submitted to the WTCRF MRC Human Genetics Unit (University of Edinburgh) for further processing. cRNA quality was checked using a Agilent 2100 Bioanalyser and hybridization performed on an MouseWG-6 v2 BeadChip (Illumina Cat # BD-201-0202). Illumina BeadArray data was analysed in R using the beadarray ([Dunning et al., 2007](#_ENREF_5)), limma ([Ritchie et al., 2015](#_ENREF_16)), and sva ([Johnson et al., 2007](#_ENREF_11)) packages. Briefly, batch-specific effects were removed from the quantile-normalized microarray data using ComBat ([Johnson et al., 2007](#_ENREF_11)) and pairwise comparisons performed between all sample groups with limma ([Ritchie et al., 2015](#_ENREF_16)). Differentially expressed genes (FDR <= 0.05 and |log2FC|>=log2(1.5)) were tested for functional enrichments using DAVID ([Huang da et al., 2009a](#_ENREF_9), [Huang da et al., 2009b](#_ENREF_10)). To juxtapose these data with our previously results ([Festuccia et al., 2012](#_ENREF_6)), the GeneProf REST web services ([Halbritter et al., 2014](#_ENREF_7)) was used to retrieve and integrate all data.

**5mC analysis by restriction digestion**

Genomic DNA was prepared from sorted E14Tg2a Esrrb-2a-GFPd-IBIH ESCs or TNG Esrrb-2a-TdTomato EpiSC using a DNeasy kit (Qiagen Cat # 69504). 1μg of chromatin diluted in 17μl of 2mM Tris/200uM EDTA (20% TE) was mixed with 2μl of the appropriate 10x restriction buffer. After transferring 9.5μl to a separate tube, which served as an undigested control, 5U of TaqI, SsiI, Hin6I (Thermo Scientific Cat # ER0671, ER1791, ER0481) or HpaII (NEB Cat # R0171L) were added to the reaction. After incubation overnight in an air oven at 37°C, 115μl of 20% TE were added to both digested and undigested samples and 5μl taken for each PCR using the oligonucleotides listed in Table EV4. PCRs were performed in technical triplicate on material from at least 4 independent biological samples.

**Supplemental References:**

Buecker C, Srinivasan R, Wu Z, Calo E, Acampora D, Faial T, Simeone A, Tan M, Swigut T, Wysocka J (2014) Reorganization of enhancer patterns in transition from naive to primed pluripotency. *Cell Stem Cell* 14: 838-53

Carpenter AE, Jones TR, Lamprecht MR, Clarke C, Kang IH, Friman O, Guertin DA, Chang JH, Lindquist RA, Moffat J, Golland P, Sabatini DM (2006) CellProfiler: image analysis software for identifying and quantifying cell phenotypes. *Genome Biol* 7: R100

Chambers I (2004) Mechanisms and factors in embryonic stem cell self-renewal. *Rend Fis Acc Lincei* s.9, v.16: 83-97

Chen X, Xu H, Yuan P, Fang F, Huss M, Vega VB, Wong E, Orlov YL, Zhang W, Jiang J, Loh YH, Yeo HC, Yeo ZX, Narang V, Govindarajan KR, Leong B, Shahab A, Ruan Y, Bourque G, Sung WK et al. (2008) Integration of external signaling pathways with the core transcriptional network in embryonic stem cells. *Cell* 133: 1106-17

Dunning MJ, Smith ML, Ritchie ME, Tavare S (2007) beadarray: R classes and methods for Illumina bead-based data. *Bioinformatics* 23: 2183-4

Festuccia N, Osorno R, Halbritter F, Karwacki-Neisius V, Navarro P, Colby D, Wong F, Yates A, Tomlinson SR, Chambers I (2012) Esrrb is a direct Nanog target gene that can substitute for Nanog function in pluripotent cells. *Cell Stem Cell* 11: 477-90

Halbritter F, Kousa AI, Tomlinson SR (2014) GeneProf data: a resource of curated, integrated and reusable high-throughput genomics experiments. *Nucleic acids research* 42: D851-8

Ho L, Jothi R, Ronan JL, Cui K, Zhao K, Crabtree GR (2009) An embryonic stem cell chromatin remodeling complex, esBAF, is an essential component of the core pluripotency transcriptional network. *Proceedings of the National Academy of Sciences of the United States of America* 106: 5187-91

Huang da W, Sherman BT, Lempicki RA (2009a) Bioinformatics enrichment tools: paths toward the comprehensive functional analysis of large gene lists. *Nucleic acids research* 37: 1-13

Huang da W, Sherman BT, Lempicki RA (2009b) Systematic and integrative analysis of large gene lists using DAVID bioinformatics resources. *Nature protocols* 4: 44-57

Johnson WE, Li C, Rabinovic A (2007) Adjusting batch effects in microarray expression data using empirical Bayes methods. *Biostatistics* 8: 118-27

Kagey MH, Newman JJ, Bilodeau S, Zhan Y, Orlando DA, van Berkum NL, Ebmeier CC, Goossens J, Rahl PB, Levine SS, Taatjes DJ, Dekker J, Young RA (2010) Mediator and cohesin connect gene expression and chromatin architecture. *Nature*

Ma Z, Swigut T, Valouev A, Rada-Iglesias A, Wysocka J (2011) Sequence-specific regulator Prdm14 safeguards mouse ESCs from entering extraembryonic endoderm fates. *Nature structural & molecular biology* 18: 120-7

Percharde M, Lavial F, Ng JH, Kumar V, Tomaz RA, Martin N, Yeo JC, Gil J, Prabhakar S, Ng HH, Parker MG, Azuara V (2012) Ncoa3 functions as an essential Esrrb coactivator to sustain embryonic stem cell self-renewal and reprogramming. *Genes & development* 26: 2286-98

Respuela P, Nikolic M, Tan M, Frommolt P, Zhao Y, Wysocka J, Rada-Iglesias A (2016) Foxd3 Promotes Exit from Naive Pluripotency through Enhancer Decommissioning and Inhibits Germline Specification. *Cell stem cell* 18: 118-33

Ritchie ME, Phipson B, Wu D, Hu Y, Law CW, Shi W, Smyth GK (2015) limma powers differential expression analyses for RNA-sequencing and microarray studies. *Nucleic Acids Res* 43: e47

Schnetz MP, Handoko L, Akhtar-Zaidi B, Bartels CF, Pereira CF, Fisher AG, Adams DJ, Flicek P, Crawford GE, Laframboise T, Tesar P, Wei CL, Scacheri PC (2010) CHD7 targets active gene enhancer elements to modulate ES cell-specific gene expression. *PLoS genetics* 6: e1001023

Wu H, D'Alessio AC, Ito S, Xia K, Wang Z, Cui K, Zhao K, Sun YE, Zhang Y (2011) Dual functions of Tet1 in transcriptional regulation in mouse embryonic stem cells. *Nature* 473: 389-93

Yang SH, Kalkan T, Morissroe C, Marks H, Stunnenberg H, Smith A, Sharrocks AD (2014) Otx2 and Oct4 drive early enhancer activation during embryonic stem cell transition from naive pluripotency. *Cell Rep* 7: 1968-81
