# Supplementary material for: Esrrb extinction triggers dismantling of naïve pluripotency and marks commitment to differentiation
Source: EMBO J. 2018 Oct 1;37(21):e95476. doi: 10.15252/embj.201695476 (PMC6213284; doi:10.15252/embj.201695476)
Supplement: Supplementary file 2 — Expanded View Figures PDF [file EMBJ-37-e95476-s002.pdf]

Expanded View Figures

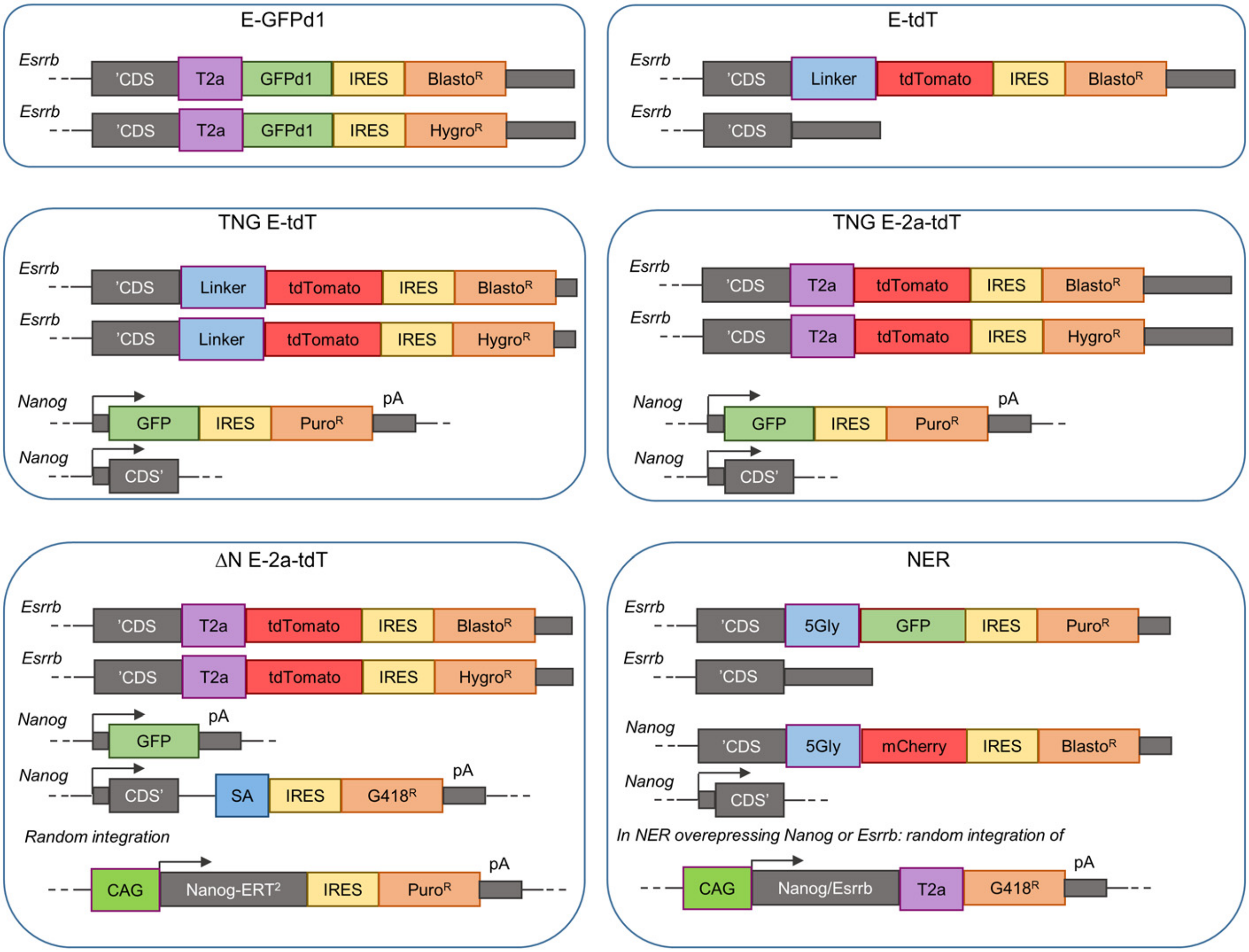

Figure EV1. Schematic representation of all ES cell lines derived in this study.
The schemes show the genetic construction of the different reporter lines used in this study. Black arrows indicate promoters. CDS', Beginning of the coding sequence; 'CDS, End of the coding sequence; 5Gly, Glycine linker; GFPd1, Destabilised GFP; BlastoR, HygroR, G418R and PuroR, Resistance genes for Basticidin, Hygromycin, G418 and Puromycin respectively; T2a, Self-cleaving linker peptides; pA, Polyadenylation signals.

© 2018 The Authors

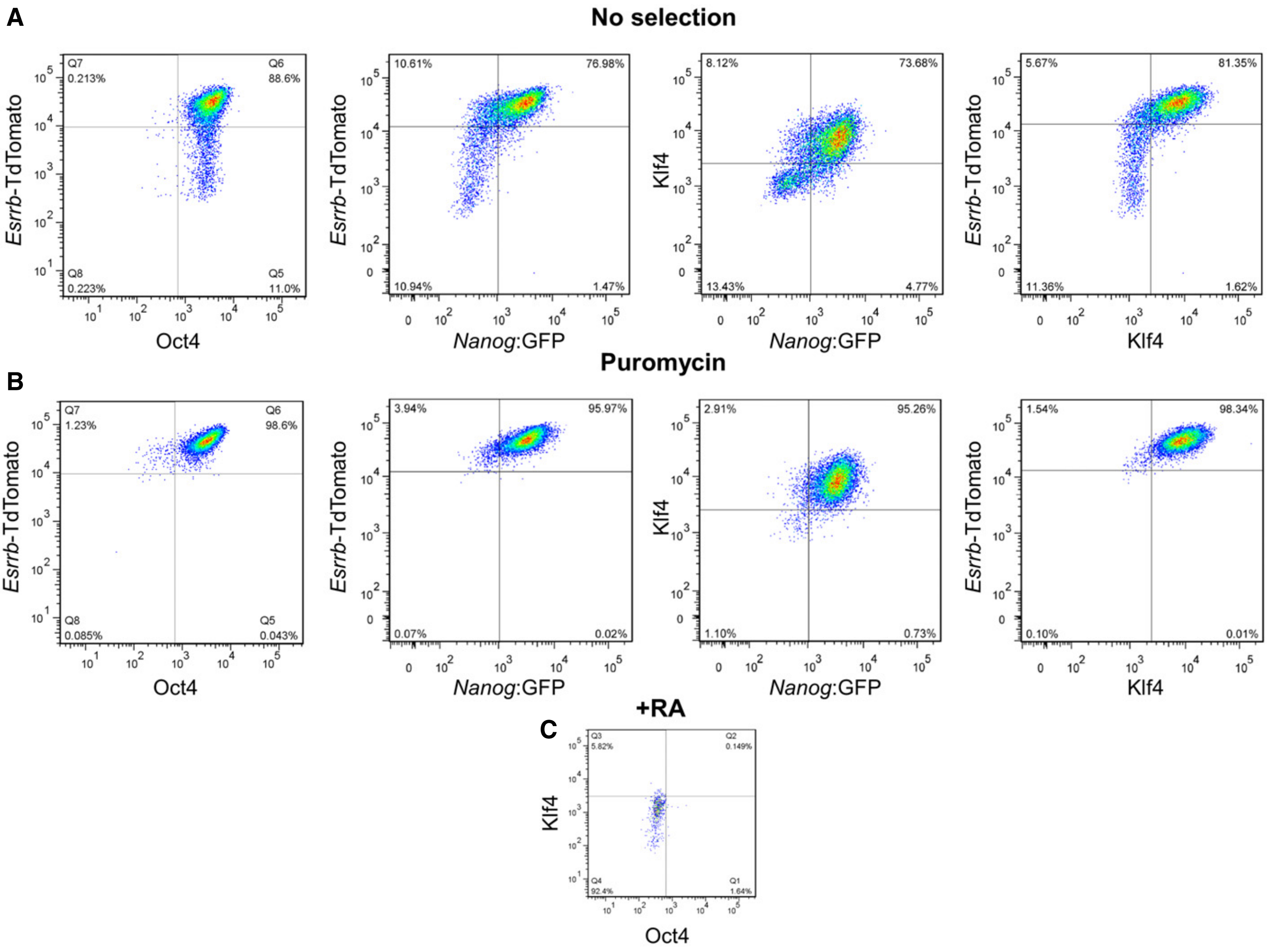

Figure EV2. NANOG controls both Esrrb and Klf4 expression in single ES cells (relates to Figs 1 and 2).

A–C TNG E-tdT ES cells were assessed by intracellular FACS for OCT4 and KLF4 (see Materials and Methods). Expression is shown relative to either Esrrb-TdTomato or Nanog:GFP after 3 days in LIF/FCS in the absence (A) or the presence (B) of selection for puromycin resistance, expressed from the Nanog:GFP allele. The levels of OCT4 and KLF4 in ESCs that had been differentiated using retinoic acid for 6 days are shown in (C).

Data information: For a schematic representation of the reporter allele configuration characteristic of each cell line, please refer to Fig EV1.

 © 2018 The Authors

Figure EV3. Distinct Esrrb compartments possess characteristic transcriptional states and differentiation propensity. Transition between these compartments is not equally reversible (relates to Fig 3).

A Kinetics of distribution of Esrrb expression levels from differentially sorted E-GFPd1 ESCs. SSEA-1^+ cells were sorted into $Esrrb^{Hi}$, $Esrrb^{Med}$, and $Esrrb^{Neg}$ gates to a purity of ≥ 99.4%. Sorted cells were then cultured in LIF/FCS for 3 days and analyzed daily. The GFP expression profile of control E14Tg2a cells is shown as a dotted line. The percentage of cells falling in the $Esrrb^{Hi}$, $Esrrb^{Med}$, or $Esrrb^{Neg}$ gates 3 days after sorting is shown for each population.

B Average binding profile of OCT4, SOX2, NANOG, KLF4, ESRRB, and MED1 to OCT4- and NANOG-bound elements in the proximity of genes differentially higher expressed (FDR ≤ 0.05, log2FC > 0) in $Esrrb^{Hi}$ than $Esrrb^{Med}$ (EH>EM, dark red) or $Esrrb^{Hi}$ than $Esrrb^{Neg}$ (EH<EN, light red) ESCs, or differentially lower expressed (FDR ≤ 0.05, log2FC < 0) in $Esrrb^{Hi}$ than $Esrrb^{Med}$ (EH<EM, dark blue) or $Esrrb^{Hi}$ than $Esrrb^{Neg}$ (EH<EN, light blue) ESCs. Data for all peaks near genes measured in our microarray assay are shown for comparison [gray dashed line; data from Chen *et al* (2008) and Kagey *et al* (2010)]. Read counts per base pair were normalized by library size and by subtracting the background signal (IgG).

C Timecourse analysis of gene expression changes upon replating sorted SSEA-1^+ $Esrrb^{Hi}$ or $Esrrb^{Neg}$ E-GFPd1 ESCs into N2B27/Activin/FGF. mRNA levels presented are relative to Tubulin beta. Error bars: standard deviation of the measures in three independent experiments (with the exception of T/Bra).

D Boxplots of expression levels of all genes showing higher (up) or lower expression (down) in epiblast/ectoderm cells from embryos dissected at cavity (CAV)/prestreak (PS) compared to late midstreak (LMS)/late streak (LS) stages. The distribution of expression levels of the two sets of differentially expressed genes is shown for $Esrrb^{Hi}$ (H), $Esrrb^{Med}$ (M), and $Esrrb^{Neg}$ (Neg) E-GFPd1 ESCs and embryo-derived (late bud stage) TNG E-2a-tdT EpiSCs (Epi). Gene expression distributions in cavity (CAV), prestreak (PS), late midstreak (LMS), late streak (LS), no bud (OB), early bud (EB), or late bud (LB) stage embryos from Kojima *et al* (2014) are shown for comparison. Boxes span the inter-quartile range (IQR) from the first to the third quartile. The line indicates the median. Whiskers extend up to 1.5 IQR, and outliers are plotted.

E Gene ontology classification of genes differentially expressed between $Esrrb^{Hi}$ and $Esrrb^{Med}$ (EH-EM), $Esrrb^{Med}$, and $Esrrb^{Neg}$ (EM-EN) and $Esrrb^{Neg}$ and EpiSCs (EN-Epi).

Data information: For a schematic representation of the reporter allele configuration characteristic of each cell line, please refer to Fig EV1.

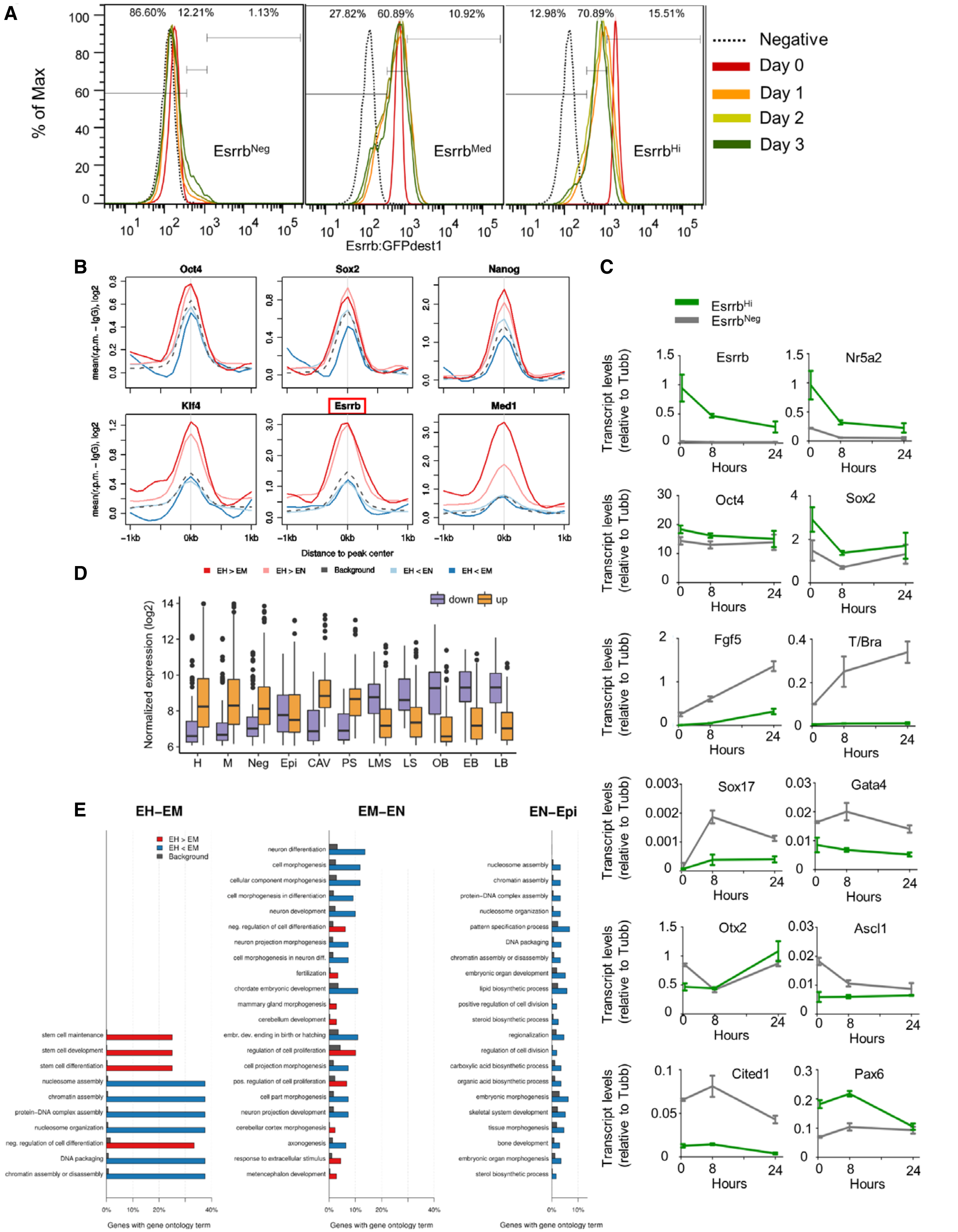
A
% of Max
86.60% 12.21% 1.13%
27.82% 60.89% 10.92%
12.98% 70.89% 15.51%
EsrrbNeg
EsrrbMed
EsrrbHi
Negative
Day 0
Day 1
Day 2
Day 3
Esrrb:GFPdest1
B
Oct4
Sox2
Nanog
Klf4
Esrrb
Med1
mean(r.p.m. − IgG), log2
Distance to peak center
EH > EM
EH > EN
Background
EH < EN
EH < EM
C
EsrrbHi
EsrrbNeg
Transcript levels (relative to Tubb)
Esrrb
Nr5a2
Oct4
Sox2
Fgf5
T/Bra
Sox17
Gata4
Otx2
Ascl1
Cited1
Pax6
Hours
D
Normalized expression (log2)
down
up
H M Neg Epi CAV PS LMS LS OB EB LB
E
EH−EM
EM−EN
EN−Epi
Genes with gene ontology term

Figure EV3.

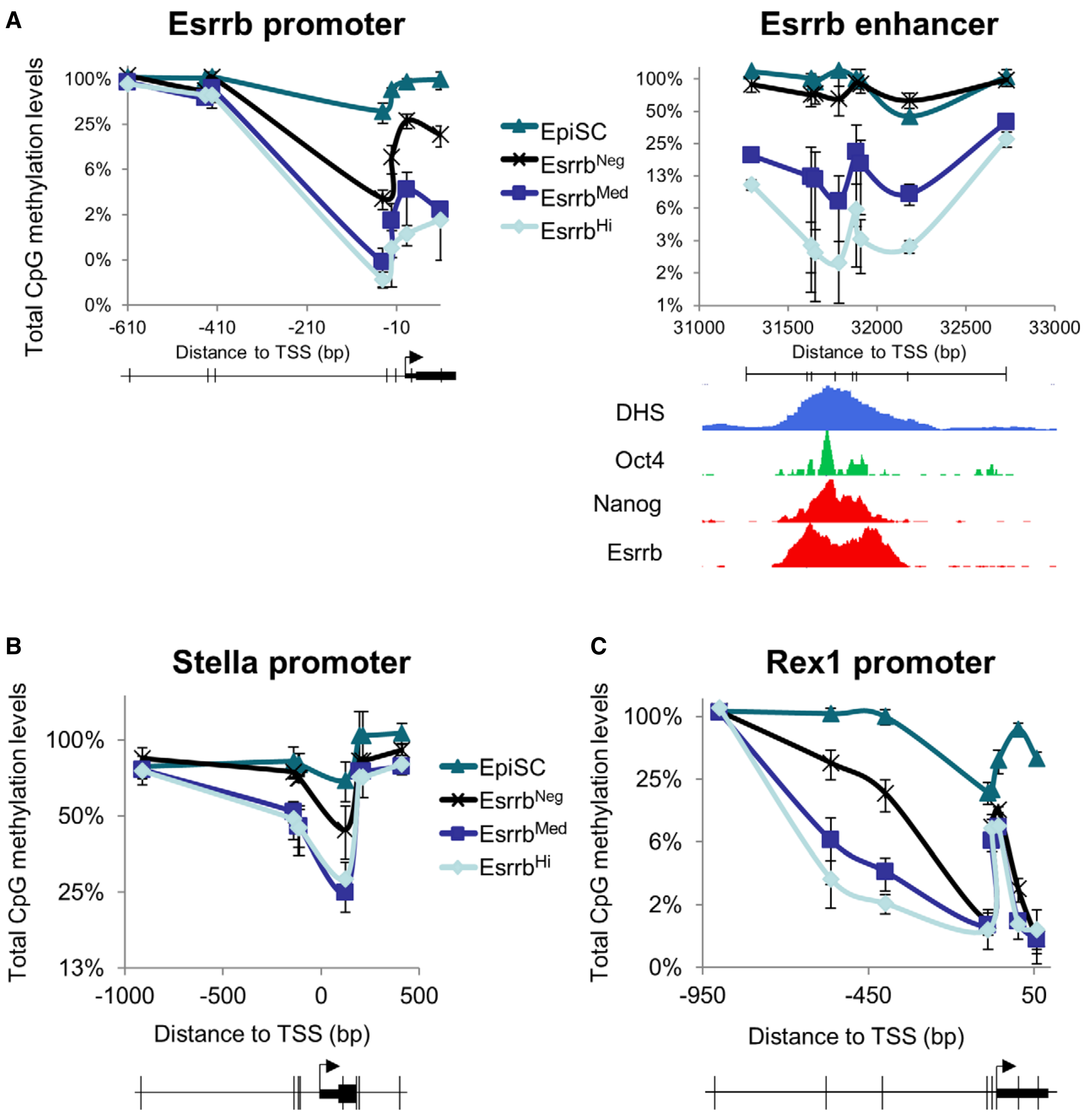

Figure EV4. Kinetics of accumulation of CpG methylation at key regulatory elements of the pluripotency gene regulatory network (relates to Fig 4).

A–C Percentage of methylated CpG dinucleotides profiled across the *Esrrb* enhancer and promoter (A), *Stella* (B), and *Rex1* (C) promoters in sorted SSEA-1^{+}/Esrrb^{Hi}, Esrrb^{Med}, or Esrrb^{Neg} E-GFPd1 ESCs and TNG E-2a-tdT EpiSC. CpG methylation was assessed by measuring protection from digestion of the *HpaII*, *AciI*, *Hin6I*, or *TaqI* restriction sites [coordinates indicated by vertical lines in the gene structure maps derived from the mouse reference genome (mm9). Locations are expressed relative to the TSS in ESCs (Chambers, 2004; Festuccia *et al*, 2012)]. Values represent total CpG methylation levels (5mC + 5hmC). Error bars: standard deviation of the measures in four independent experiments. ChIP-Seq read coverage for the indicated factors derived from re-analysis of published datasets (Chen *et al*, 2008) shows how TF binding correlates with methylation "valleys" and increased chromatin accessibility. RNA polymerase II binding marks promoters (Stadler *et al*, 2011). The DNase I sensitivity in E14 ES cells data is from the ENCODE project.

© 2018 The Authors

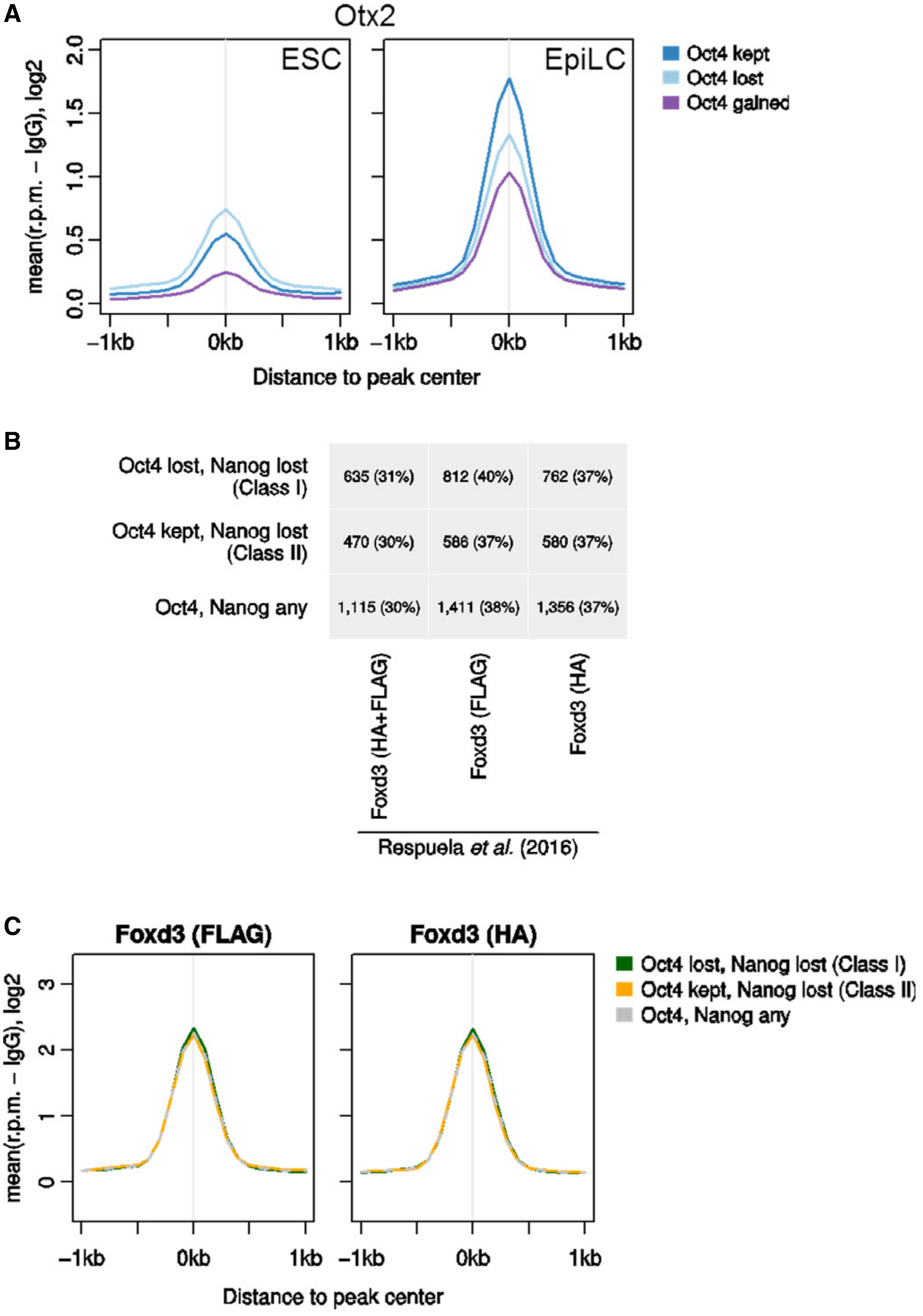

Figure EV5. FOXD3 and OTX2 do not act differentially on Class I or Class II regions during commitment (relates to Fig 6).

A Average ChIP-Seq profiles for OTX2 in ESCs or EpiLC at OCT4 peaks that are kept, lost, or gained in $Esrrb^{Neg}$ relative to $Esrrb^{Hi}$ ESCs [data from Buecker *et al* (2014)].

B Overlap of Class I, Class II, or any OCT4/NANOG-bound element (top to bottom) with FOXD3-bound elements identified in ESC by ChIP-Seq with antibodies against HA (right), FLAG (center), or the bioinformatic intersection of both peak sets (left). Percentages are the number of overlapping peaks relative to the total number of Class I (top row), Class II (middle row), or all OCT4/NANOG-bound peaks [bottom row; data from Respuela *et al* (2016)].

C Average binding profile of FOXD3 using FLAG (left) and HA (right) antibodies to Class I (green), Class II (orange), or all Nanog- and Oct4-bound (gray dashed line) elements in ESCs [data from Respuela *et al* (2016)]. Read counts per base pair were normalized by library size and by subtracting the background signal (IgG).

 © 2018 The Authors
